# Low-dose acetylsalicylic acid reduces local inflammation and tissue perfusion in dense breast tissue in postmenopausal women

**DOI:** 10.1186/s13058-024-01780-2

**Published:** 2024-02-05

**Authors:** Peter Lundberg, Annelie Abrahamsson, Johan Kihlberg, Jens Tellman, Ieva Tomkeviciene, Anette Karlsson, Maria Kristoffersen Wiberg, Marcel Warntjes, Charlotta Dabrosin

**Affiliations:** 1 Department of Radiation Physics and Department of Medical and Health Sciences, Linköping University, Linköping, Sweden; 2 Center for Medical Image Science and Visualization (CMIV), Linköping University, Linköping, Sweden; 3 Department of Oncology and Department of Biomedical and Clinical Sciences, Linköping University, 581 85 Linköping, Sweden; 4 Department of Radiology and Department Medical and Health Sciences, Linköping University, Linköping, Sweden

**Keywords:** Randomized trial, MRI, Mammography, Inflammation, Microdialysis

## Abstract

**Purpose:**

One major risk factor for breast cancer is high mammographic density. It has been estimated that dense breast tissue contributes to ~ 30% of all breast cancer. Prevention targeting dense breast tissue has the potential to improve breast cancer mortality and morbidity. Anti-estrogens, which may be associated with severe side-effects, can be used for prevention of breast cancer in women with high risk of the disease per se. However, no preventive therapy targeting dense breasts is currently available. Inflammation is a hallmark of cancer. Although the biological mechanisms involved in the increased risk of cancer in dense breasts is not yet fully understood, high mammographic density has been associated with increased inflammation. We investigated whether low-dose acetylsalicylic acid (ASA) affects local breast tissue inflammation and/or structural and dynamic changes in dense breasts.

**Methods:**

Postmenopausal women with mammographic dense breasts on their regular mammography screen were identified. A total of 53 women were randomized to receive ASA 160 mg/day or no treatment for 6 months. Magnetic resonance imaging (MRI) was performed before and after 6 months for a sophisticated and continuous measure breast density by calculating lean tissue fraction (LTF). Additionally, dynamic quantifications including tissue perfusion were performed. Microdialysis for sampling of proteins in vivo from breasts and abdominal subcutaneous fat, as a measure of systemic effects, before and after 6 months were performed. A panel of 92 inflammatory proteins were quantified in the microdialysates using proximity extension assay.

**Results:**

After correction for false discovery rate, 20 of the 92 inflammatory proteins were significantly decreased in breast tissue after ASA treatment, whereas no systemic effects were detected. In the no-treatment group, protein levels were unaffected. Breast density, measured by LTF on MRI, were unaffected in both groups. ASA significantly decreased the perfusion rate. The perfusion rate correlated positively with local breast tissue concentration of VEGF.

**Conclusions:**

ASA may shape the local breast tissue microenvironment into an anti-tumorigenic state. Trials investigating the effects of low-dose ASA and risk of primary breast cancer among postmenopausal women with maintained high mammographic density are warranted.

*Trial registration* EudraCT: 2017-000317-22.

**Supplementary Information:**

The online version contains supplementary material available at 10.1186/s13058-024-01780-2.

## Introduction

Breast cancer affects more than 10% of all women in the Western world, and its incidence continues to rise [1]. Although mammography screening programs and improved treatments have reduced the death rate of breast cancer, efficient prevention strategies could further reduce the mortality and morbidity associated with this disease [1]. One major risk factor for breast cancer is increased mammographic density [2]. Women with > 75% dense breast tissue have a fourfold increased risk of developing breast cancer compared with women with < 5% dense breast tissue [2, 3]. Additionally, dense breast tissue is estimated to contribute to ~ 30% of all breast cancer [4].

Anti-estrogens, such as tamoxifen and aromatase inhibitors, may reduce the risk of breast cancer per se by 50% not taking breast density into consideration [5, 6]. These therapies may, however, have severe side-effects, such as osteoporosis, endometrial cancer, thromboembolism, and decreased quality of life, including vaginal atrophy and sexual dysfunction. Because of this the adherence to these types of therapies is very low. Thus, there is a need for novel preventive strategies and therapeutics for women with dense breasts.

An inflammatory microenvironment, being a hallmark of cancer, is associated with an increased risk of breast cancer [7]. We and others show that dense breast tissue is associated with increased inflammation [8–10]. Additionally, several studies report that the risk of breast cancer may be affected by the regular use of anti-inflammatory drugs [11–21]. In four large systematic reviews and meta-analyzes including up to 2,000,000 women, acetylsalicylic acid (ASA) and non-steroidal anti-inflammatory drug (NSAID) use were associated with a ~ 20% reduced risk of breast cancer [15–17, 20]. However, two large prospective cohort studies failed to find an association between NSAID use and breast cancer risk [22, 23].

Very few studies have investigated the possible effect of anti-inflammatory drugs on mammographic density. In one study, use of NSAIDs was not associated with breast density in a population of 3000 women. In a randomized trial including postmenopausal women, a daily dose of 325 mg ASA for 6 months did not affect mammographic density [24, 25]. However, most women in the trial had breast densities corresponding to B or C on the Breast Imaging Reporting and Data System (BI-RADS) scale, whereas less than 10% had Bi-RADS D breast density, indicating extremely dense tissue [25]. Thus, studies of the possible effect of ASA in women within the highest breast density category are lacking. Moreover, the local biological effects of ASA in dense breast tissue have yet to be elucidated, and studies using sophisticated measures to assess structural and dynamic changes after treatment have not yet been performed.

This study aimed to determine whether delivering low-dose ASA to postmenopausal women with dense breast tissue would affect the inflammatory microenvironment and magnetic resonance imaging (MRI) parameters in the breast. We recruited healthy postmenopausal women from a mammography screening program categorized as having BI-RADS D breast density. Women underwent MRI to assess structural and dynamic characteristics, including continuous breast density quantification, perfusion quantification, and calculation of apparent diffusion coefficient (ADC) and relaxometric parameters. For biological characterization, microdialysis was performed to measure inflammatory proteins locally in breast tissue and from abdominal subcutaneous fat as a measure of systemic effects. Women were randomized to receive ASA 160 mg/day for 6 months or no treatment. We found that ASA therapy for 6 months resulted in a less inflammatory breast tissue microenvironment and decreased tissue perfusion.

## Materials and methods

### Participants

The Regional Ethical Review Board of Linköping, Sweden approved the study protocol, which was carried out in accordance with the Declaration of Helsinki (Swedish Medical Product Agency with the Clinical Trial Number EudraCT: 2017-000317-22, registered April 13, 2017, and Regional Ethical Review Board, Dnr 2017/111-31, approved April 10, 2017). All women gave written informed consent.

Healthy postmenopausal women (55–74 years of age) from the mammography screening program at Linköping University Hospital who were categorized as having BI-RADS D (i.e.,,, extremely dense) breast tissue according to the Breast Imaging Reporting and Data System [26] were invited to participate in the study. Inclusion criteria were postmenopausal, defined as more than 12 months of amenorrhea; a baseline breast density score of BI-RADS D for the left breast; no serious co-morbidity; willingness to take ASA for 6 months; and willingness to avoid regular intake of NSAIDs outside of the trial. Exclusion criteria were any sex steroid use within the last 3 months, including systemic hormone replacement therapy; use of contraceptives, including hormonal intrauterine devices; use of anti-estrogen therapy, including selective estrogen modulators or degraders and aromatase inhibitors; previous interventions of the breast; current regular use of NSAIDs; known intolerance to or contraindications for NSAIDs; diabetes; current smoking; uncontrolled hypertension; or any contraindication for MRI, including allergy to contrast agents. Multimodal MRI followed by microdialysis was performed for all women at baseline and after 6 months.

### MRI data acquisition and analysis

A 3 T Ingenia MRI scanner (Philips Healthcare, Best, The Netherlands) with a dual-breast 16-element breast coil was used for MRI data acquisition. Co-localization and analysis of lean tissue fraction (LTF) and proton density fat fraction (PDFF), diffusion-weighted images (DWI), and perfusion dynamics were performed using Matlab (Version 2020b, The Mathworks Inc, Natick, Massachusetts, USA). Quantitative relaxometry (qMRI) was also performed.

### Magnetic resonance spectroscopy

A magnetic resonance spectroscopy (MRS) voxel (i.e.,,, volume of interest [VOI]) with a minimum size of 10 × 10 × 10 mm^3^ (but up to 20 mm side length in some participants) was located in the upper lateral quadrant of the left breast, which was the same location as the microdialysis catheter. MRS data were acquired with TR 2000 ms and TE 35, 70, 140, and 180 ms with PRESS as the volume selection method in 16 averages. Post-processing was performed using LCModel (http://s-provencher.com/lcmodel.shtml).

Data from different acquisitions (i.e.,,, perfusion, PDFF, LTF, and diffusion) were extracted from the same VOI as was used for MRS using eTHRIVE pre-contrast agent injection. Volumetric and positional information from image metadata were used to find the corresponding voxel coordinates of the VOI for perfusion, LTF, and DWI volumes, respectively.

### Lean tissue fraction

Axial 3D four-point echo turbo field MRI images were acquired with anterior–posterior frequency encoding using an initial TE 1.15 ms and ΔTE 1.15 ms, TR 10 ms, flip angle 10°, field of view (FoV) 300 × 316 mm^2^, 120 × 127 scan matrix, and 1.8 mm slice thickness. Data acquisition was performed separately before and after intravenous contrast agent injection.

Water- and fat-separated MRI images were constructed using four echoes [27], and LTF maps were constructed as the ratio of lean tissue volume to total volume. Quantitative fat images were computed by calibrating the original fat images using adipose tissue as an internal intensity reference, which also allowed adipose tissue volume to be quantified within the segmentation [28, 29]. LTF data were extracted from LTF images within the MRS VOI.

### Diffusion measurements

Diffusion data were obtained using an echo-planar (EPI) single shot DWI with a TR/TE 11,671/91 ms, EPI factor 75, three averages, fat suppression with 'SPectral Attenuated Inversion Recovery' (SPAIR), FoV 300 × 317 mm^2^, slice thickness 3.0 mm, gap 0.3 mm, resolution 2.2 × 2.2 mm^2^ (recon 1.0 × 1.0 mm^2^), and b-values 0, 100, and 850 s/mm^2^. ADC maps were then computed using manufacturer-provided MRI scanner software. ADC data were extracted from ADC images within the MRS VOI.

### Perfusion measurements

Perfusion data were obtained from a 3D spoiled gradient echo with TR/TE 2.5/1.27 ms, flip angle 12°, matrix 136 × 140, FoV 300 × 309 mm^2^, recon resolution 1.0 × 1.0 mm^2^, 4.4 mm slice thickness, and -2.2 mm gap. Sixty dynamics were collected during 3:10 min and five dynamics after 7 min, with each dynamic lasting 3 s. Contrast injection started at the same time as perfusion sequences at a rate of 2 mL/s, resulting in six or seven dynamics without contrast media. The contrast medium was 10 mL gadoteric acid (Clariscan®, GE Healthcare Limited, Little Chalfont, England) followed by 30 mL saline using a MEDRAD® MRXperion injector (Bayer HealthCare, Whippany, NJ, USA).

Perfusion characteristics were measured using both a mathematical fitting procedure on the entire MRS VOI and a blinded visual review procedure by radiologists in a selected single slice in the same location as the MRS VOI.

*Mathematical modeling.* Mathematical fitting was performed by extracting temporal data from the entire MRS VOI. To calculate the area under the perfusion curve (AUC) and time constant (tau), the mean intensity values within each temporal VOI were normalized to baseline values and applied to all acquisitions in the perfusion series. Normalized data points were fitted in Matlab using a non-linear least-squares algorithm as previously described [27].

*Radiological review.* Representative time points were selected through the region of interest in the upper lateral quadrant of the left breast just before the perfusion curve peaked (usually around 120 s (i.e.,,, “wash-in”)) and after the curve peaked (usually around 300 s (i.e.,,, “wash-out”)). If the curve was constantly rising, times around 120 s and 300 s were chosen. Wash-in and wash-out were expressed as the signal increase (%) relative to baseline intensity. This procedure was performed using IntelliSpace Portal (ISPv11.0, Philips Healthcare, Best, Netherlands).

### Relaxometry

Relaxometry (and synthetic MRI) was performed using 3D-QALAS [30], a multi-dynamic 3D gradient multi-echo sequence with TR/TE 5.0/2.3 ms, matrix 179 × 178, FoV 300 × 320 mm^2^, and slice thickness 1.8 mm. Quantitative R1 and R2 relaxation rates and proton density PD maps were generated by SyMRI (SyntheticMR, Linköping, Sweden). Entire breasts were segmented from the whole acquisition volume, but an additional 5 mm was removed from the edges to suppress the contribution of skin tissue. The images were further segmented based on selected ranges in quantitative R1, R2, and PD into fibroglandular tissue, fat, and remaining tissue including milk ducts and blood vessels. Based on the quantitative R1, R2 and PD maps synthetic T1-, T2- and PD-weighted images were reconstructed.

### Conventional imaging sequences

Conventional clinical sequences for diagnostics were also acquired, such as STIR (TR/TE 4370/65 ms, inversion time 240 ms, matrix 300 × 253, FoV 300 × 320 mm^2^, slice thickness 3.0 mm, gap 0.3 mm, recon resolution 0.7 × 0.7 mm^2^), T2 3D (TR/TE 2000/308 ms, matrix 378 × 397, FoV 300 × 317 mm^2^, slice thickness 2.0 mm, gap -1.0 mm, recon resolution 0.7 × 0.7 mm^2^), and T1 3D fat suppressed (TR/TE 5.9/2.9 ms, matrix 500 × 531, FoV 300 × 317 mm^2^, slice thickness 1.0 mm, gap -0.5 mm, recon resolution 0.5 × 0.5 mm^2^).

### Microdialysis procedure

Prior to the insertion of microdialysis catheters, 0.5 mL lidocaine (10 mg*/*mL) was administrated intracutaneously. Microdialysis catheters (M Dialysis AB, Stockholm, Sweden), which consisted of a 20-mm-long tubular dialysis membrane (diameter 0.52 mm, 100,000 atomic mass cut-off) glued to the end of a double-lumen tube, were inserted via a splittable introducer (M Dialysis AB) connected to a microinfusion pump (M Dialysis AB) and perfused with 154 mmol*/*L NaCl and 60 g/L hydroxyethyl starch (Voluven®; Fresenius Kabi, Uppsala, Sweden) at 0.5 µL/min. One catheter was placed in the upper lateral quadrant of the left breast directed toward the nipple, and one catheter was placed in abdominal subcutaneous (s.c.) fat as previously described [31–39]. After a 60-min equilibration period, outgoing perfusate was stored at − 80 °C for subsequent analysis.

### Protein quantification

Microdialysis samples were analyzed using multiplex proximity extension assay (Olink Bioscience, Uppsala Sweden) as previously described [40–42]. Briefly, 1 μL sample was incubated with proximity antibody pairs tagged with DNA reporter molecules. The DNA tails formed an amplicon by proximity extension, which was quantified by high-throughput real-time PCR (BioMark™ HD System; Fluidigm Corporation, South San Francisco, CA, USA). The generated fluorescent signal correlates with protein abundance by quantitation cycles (Cq) produced by BioMark Real-Time PCR software. To minimize variation within and between runs, data were normalized using both an internal control (i.e.,,, extension control) and an interplate control and transformed using a predetermined correction factor. Pre-processed data were provided in the arbitrary unit normalized protein expression (NPX) on a log_2_ scale, which was then linearized using the formula 2^NPX^. A high NPX value corresponds to a high protein concentration. Values represent relative quantification, meaning that absolute levels between different proteins cannot be compared.

### Statistical analyzes

This trial was primarily designed to test the hypothesis that in vivo extracellular levels of inflammatory proteins decrease in women treated with ASA compared with untreated women. For sample size calculations, an effect size of 0.8 for IL-6, IL-8, and CCL5 was assumed based on mean and standard deviations from previous reports [9]. Considering a power of 0.8 and a significance level of 0.05, 25 participants per treatment arm were required. Secondary MRI outcomes were analyzed using an exploratory approach. Data were analyzed using paired Student’s t-tests, and correlations were calculated using Spearman’s rho. Proteomic data were analyzed using the two-stage set-up method of Benjamini, Krieger, and Yekutieli with a false discovery rate (FDR) set at 5%. A *p* < 0.05 was considered statistically significant. Statistical analyzes were performed with Prism 9.0 (GraphPad Software, USA).

## Results

### Study participants

**Fig. 1:**
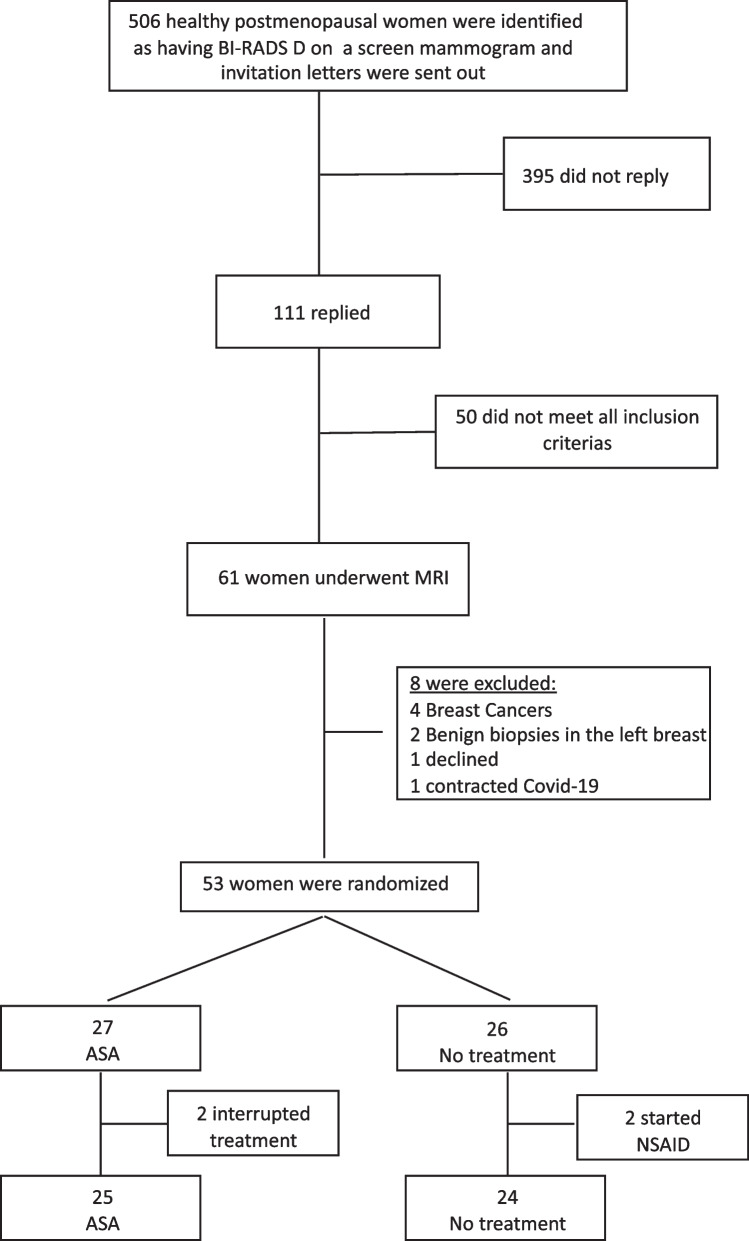
Participant flow. Strobe diagram depicting participant recruitment and inclusion/exclusion

A total of 506 women were invited to participate in the study after being identified as having BI-RADS D breast density between May 2017 and March 2020. Of the 111 women who responded and were interested in participating in the study, 61 met the study inclusion criteria (Fig. 1). After undergoing breast MRI, 21 women had atypical findings and underwent ultrasound examination. Four women were subsequently diagnosed with breast cancer and excluded from the study. Two additional women underwent biopsies of the left breast and received a benign diagnosis; these women were also excluded from the study one woman declined participation after ultrasound examination, and one contracted COVID-19 and could not participate. The remaining 13 women had benign ultrasound findings and were included in the study. Participant characteristics are shown in Additional file 1: Table S1.

A randomization list and sealed randomization envelopes were generated by a contracted independent party. After the microdialysis investigations the study subjects were randomized by consecutive opening of the envelopes. A total of 53 women underwent microdialysis and were randomized to receive ASA 160 mg/day (*n* = 27) or no treatment (*n* = 26). In the ASA group, two women interrupted treatment due to skin rash. In the no-treatment group, two women started regular use of NSAIDs for other medical reasons during the study period.

The analyzes were performed in a per-protocol population that was defined as all subjects that completed both of their microdialysis samplings, at inclusion and after 6 months and, if randomized to treatment, took at least 85% of the study drug. Data from subjects that did not fulfill the per-protocol definition were not included in the primary analysis.

Additionally, subjects developing other conditions requiring treatment with ASA, NSAID’s or anti-inflammatory drugs were withdrawn from study. Thus, 25 women in the ASA group and 24 women in the no-treatment group were included in the analyzes.

### Low-dose ASA decreased proinflammatory protein levels in breast tissue

**Fig. 2:**
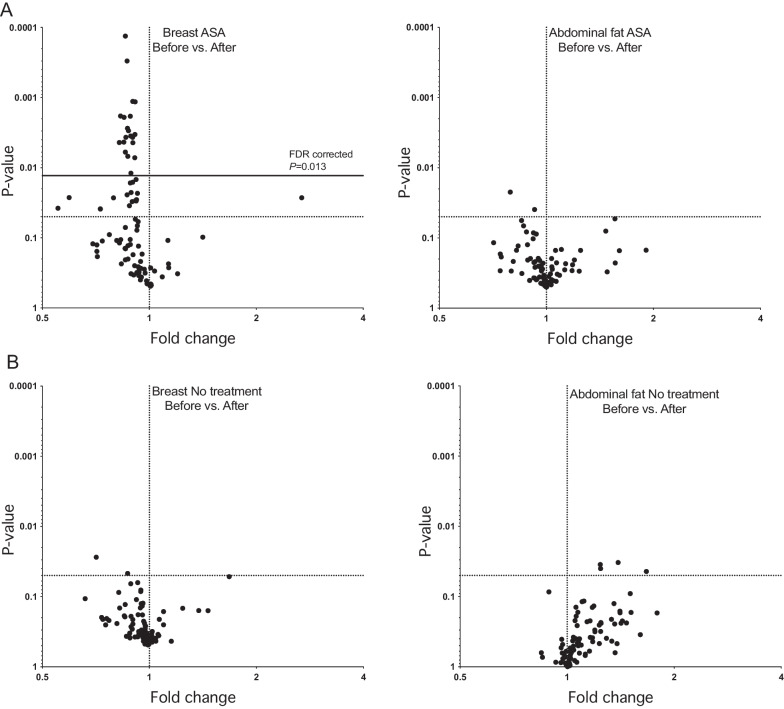
Low-dose ASA decreased levels of proinflammatory proteins in breast tissue*.* Postmenopausal healthy women categorized as having BI-RADS D breast tissue were recruited from a regular mammography screening program and randomized to receive ASA 160 mg/day or no treatment for 6 months. Microdialysis was performed directly after multimodal MRI measurements to sample inflammatory proteins locally in the left breast and abdominal s.c. fat as a measure of systemic effects before and after treatment. A panel of 92 inflammatory proteins were quantified in microdialysis samples using proximity extension assay. Proteomic data were analyzed using the two-stage set-up method of Benjamini, Krieger, and Yekutieli with a FDR of 5%. Dotted horizontal line represents *p* < 0.05. Bold line represents FDR-corrected *p* < 0.013. **A** Volcano plot for women randomized to receive ASA 160 mg/day for 6 months. **B** Volcano plot for women randomized to receive no treatment for 6 months

As the primary objective of the study was to examine changes in inflammatory proteins after ASA treatment, we analyzed microdialysis samples using a panel of 92 inflammatory proteins (Additional file 1: Table S2). Out of these 92 proteins, 20 showed decreases in breast tissue after 6 months of ASA therapy (Fig. 2A). The magnitude of changes in levels of proteins that were significantly altered in breast tissue after FDR correction ranged from 9 to 18%. Surprisingly, none of the proteins that were affected in breast tissue were altered in abdominal s.c. fat (Fig. 2A). In the no-treatment group, no changes in protein levels were observed in breast tissue or abdominal s.c. fat after 6 months (Fig. 2B).

**Fig. 3:**
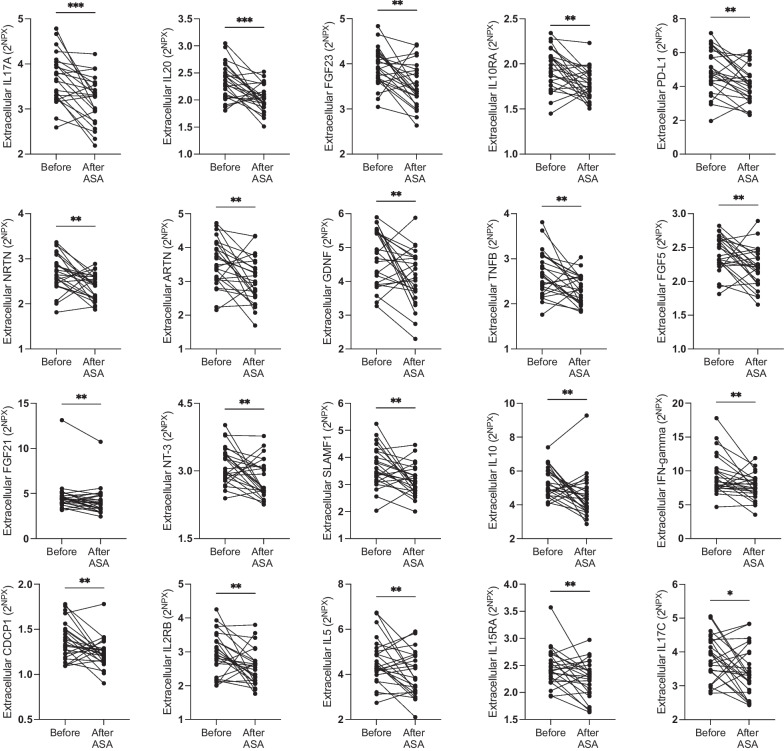
Low-dose ASA decreased levels of 20 inflammatory proteins. Postmenopausal healthy women categorized as having BI-RADS D breast tissue were recruited from a regular mammography screening program and randomized to receive ASA 160 mg/day or no treatment for 6 months. Microdialysis was performed to sample inflammatory proteins locally in the left breast before and after treatment. A panel of 92 inflammatory proteins were quantified in the microdialysis samples using proximity extension assay. Out of these 92 proteins, 20 were significantly decreased in breast tissue. *p < 0.05, **p < 0.01, ***p < 0.001

The 20 proteins with significantly altered levels in breast tissue after ASA treatment following FDR correction are depicted in Fig. 3. An additional 17 proteins in breast tissue showed changes after 6 months of ASA with a *p* < 0.05 although not after FDR correction, including decreases in TRANCE, IL-2, CD244, IL-22RA1, LIF-R, SCF, IL-1a, VEGF, IL-10RB, IL-20RA, IL-7, TNFRSF9, IL-24, CL20, CSF-1, and STA1A1 and increases in MMP-1. Levels of IL-7 and IL-13 were lower in abdominal s.c. fat with a *p* < 0.05 but not after FDR correction.

In the no-treatment group after 6 months, MCP-3 and IL-20 levels were lower in breast tissue, and TRAIL, IL-1a, MMP-10, and CXCL10 levels were higher in abdominal s.c. fat with a *p* < 0.05 but not after FDR correction.

### Low-dose ASA reduced breast tissue perfusion but did not change LTF or PDFF

**Fig. 4:**
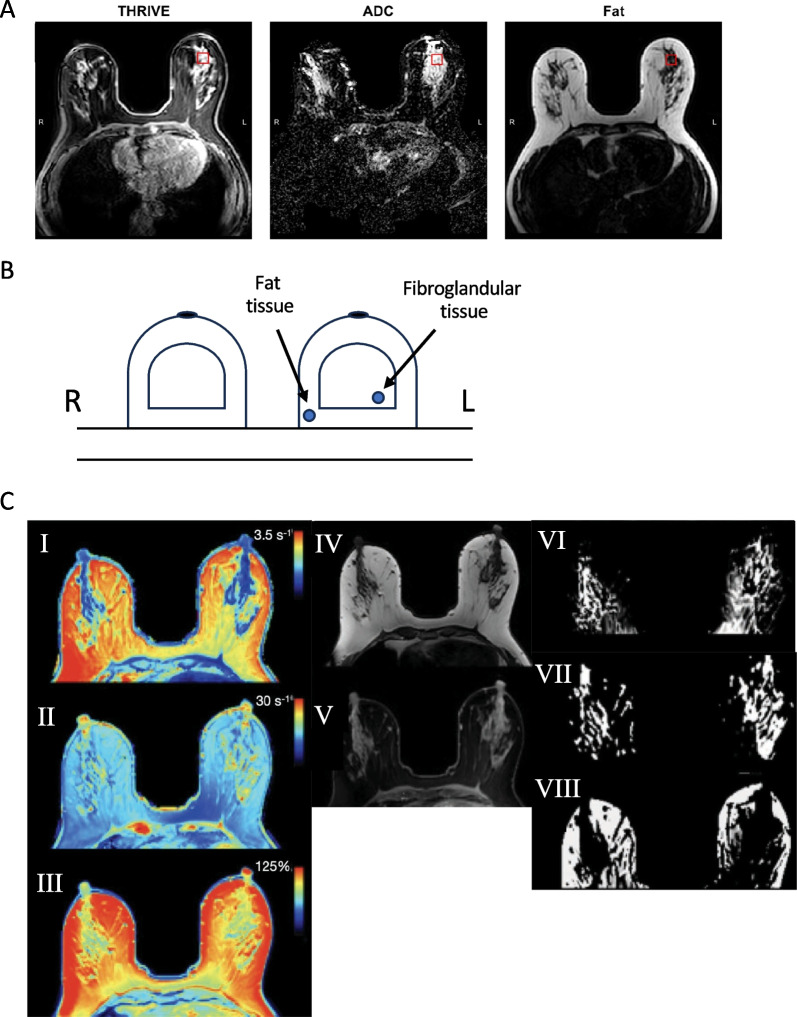
Typical images of multimodal MRI and qMRI maps*.* **A** THRIVE pre-contrast agent injection images used for perfusion assessment, ADC maps, and fat-separated images used to calculate LTF. **B** Axial breast images. Typical schematic placement of regions of interest (ROIs) in axial breast images. Blue circles show the position of ROIs placed in fibroglandular and fat tissue of the left breast for radiological measurements. **C** (I) R1 relaxation rate map; (II) R2 relaxation rate map; (III) PD map for one participant; synthetic MRI: (IV) synthetic T1w, (V) synthetic PDw-STIR (based on qMRI maps); automatic breast tissue segmentation based on qMRI-data: (VI) fibroglandular tissue, (VII) ducts and blood vessels, (VIII) fat

**Fig. 5:**
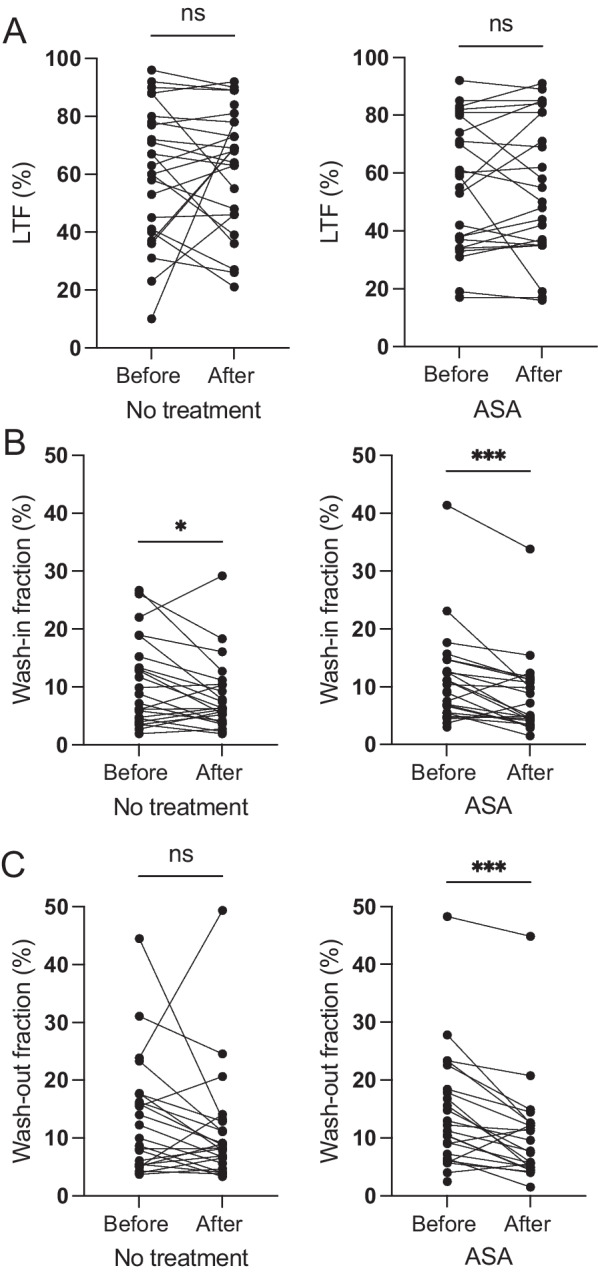
Low-dose ASA did not affect LTF but decreased perfusion rate. Postmenopausal healthy women categorized as having BI-RADS D breast tissue were recruited from a regular mammography screening program and randomized to receive ASA 160 mg/day or no treatment for 6 months. MRI was performed before and after treatment. **A** LTF was calculated in the upper lateral quadrant of the left breast as depicted in Fig. 4A. No significant changes were detected in either group. **B** Perfusion rate. A. Wash-in fraction at 120 s (% of baseline). **C** Wash-out fraction at 300 s (% of baseline). *P < 0.05, ***P < 0.001, ns = not significant

Typical examples of MRI and qMRI images used for quantifications are shown in Fig. 4. To examine breast density, we used the sophisticated continuous MRI measure of LTF before and after ASA treatment or no treatment for 6 months within the selected MRS VOI. No differences were found in either group, either with or without Gd^3+^-containing contrast agent (Fig. 5A). In addition, no significant changes were observed in PDFF. These results indicate that no significant structural changes in breast tissue occurred.

Next, we measured perfusion of breast tissue to identify possible changes in tissue physiology due to time or low-dose ASA. Two methods of data analysis were used: a within-slice method (2D) and an MRS-voxelized method (3D). The fraction of contrast agent in the wash-in phase, compared with baseline, decreased significantly after no treatment as well as after low-dose ASA (*p* < 0.001; Fig. 5B). However, significantly more women in the ASA group exhibited a decreased wash-in fraction than in the no-treatment group (*p* < 0.05, Fisher’s exact test). The wash-out fraction did not change in the no-treatment group but decreased significantly in the ASA group (*p* < 0.001; Fig. 5C). The wash-out rate correlated significantly with the potent angiogenic factor VEGF (*p* < 0.01, Spearman’s *r* = 0.359). Perfusion measured by AUC or tau did not change in either group, and no changes in ADC diffusion control measurements were found in either group (Table 1).

**Table 1:** Results of the MR-measurements

Magnetic resonance parameter	Baseline No treatment	6 Months No treatment	Baseline -ASA	6 Months + ASA
*Structural features*				
PDFF (%) mean ± SD	37 ± 22	40 ± 23	47 ± 23	43 ± 20
ADC (10^–3^ mm^2^ / s) mean ± SD	1.39 ± 0.41	1.28 ± 0.43	1.40 ± 0.37	1.38 ± 0.39
ADC Kurtosis (-) mean ± SD	3.38 ± 1.54	3.03 ± 0.99	3.01 ± 0.99	3.00 ± 0.61
*Dynamic characteristics*				
Perfusion, AUC (a.u.) mean ± SD	29 ± 20	26 ± 23	30 ± 21	25 ± 17
Perfusion, *tau* (s)	68 ± 23	70 ± 33	69 ± 30	73 ± 25
R1 (s^−1^; fibroglandular, whole breast)	2.0 ± 0.2	2.0 ± 0.2	2.0 ± 0.2	2.0 ± 0.3
R2 (s^−1^; fibroglandular, whole breast)	13.6 ± 0.5	13.5 ± 0.5	**13.7 ± 0.5**	13.4 ± 0.4*
PD (%; fibroglandular, whole breast)	84 ± 4	84 ± 3.9	85 ± 3.4	85 ± 3.5

The MR-protocol was divided into (i) measurements of structural features (ADC and PDFF), and (ii) dynamic characteristics on the tissue microstructure scale. Measurements were obtained in MRS voxel in upper left quadrant, left breast, alternatively in entire breast (for qMRI-measurements)

*PDFF* Proton Density Fat Fraction, *AUC* Area Under (perfusion curve), tau, perfusion time constant, R1, longitudinal relaxation rate, R2, transverse relaxation rate, *PD* Proton Density

*p < 0.05

### Low-dose ASA changed relaxation properties in fibroglandular tissue

There were no differences in the relaxation properties of R1, R2, or PD in fat tissue or duct and blood vessel segments. However, in the fibroglandular tissue segment, relaxation in R2 was slightly, but significantly, lower in women treated with ASA for 6 months, with a decrease from 13.7 ± 0.5 to 13.4 ± 0.4 s^−1^ (*p* < 0.05), whereas relaxation parameters were not significantly changed in women with no treatment (Table 1).

## Discussion

Breast screening programs aim to achieve early detection of already-developed cancers. For women with increased breast density, regular mammography has lower sensitivity and specificity for detecting cancer as compared with women with non-dense breasts. However, mammography can accurately determine high mammographic density and thereby identify women with increased risk of cancer. Today, interventions aimed at preventing breast cancer in this high-risk group are unavailable but have the potential to increase survival and reduce morbidity due to the disease. Anti-estrogen therapies reduce the risk of breast cancer by 30–50% without taking breast density into account [43]. However, these treatments can have severe side effects, including endometrial cancer, osteoporosis, and decreased quality of life [43, 44]. The side-effects can be reduced by lowering the dose of tamoxifen from 20 to 5 mg daily with preserved effects on decreased breast cancer risk [45]. However, it has been reported that up to 25% of postmenopausal women describe sexual dysfunction and vasomotor symptoms even after 2.5–5 mg tamoxifen daily [46]. Therefore, less toxic prevention therapies for high-risk women should be developed.

Large epidemiological studies show that regular use of anti-inflammatory drugs is associated with altered risk of breast cancer [11–21]. However, the type of anti-inflammatory drugs may be important for beneficial effects, as it is reported that ASA, but not other NSAIDs, is associated with a lower risk of breast cancer [13, 19]. Women with a family history of breast cancer and those with a personal history of benign breast disease, but not the general population, may benefit from such treatments [13, 19]. Age may be another factor, as any NSAID use is reported to reduce the risk of breast cancer in premenopausal women but not in postmenopausal women [14, 18]. However, one major confounding factor in these studies is that postmenopausal women who used ASA regularly were more likely to use sex steroids as menopausal hormone therapy [23]. Regarding use of NSAIDs after a diagnosis of breast cancer, self-reported ASA use, both pre- and post-diagnosis, is associated with a decreased risk of distant recurrence and death [47, 48]. These results are supported by a meta-analysis showing that NSAID and ASA use after, but not before, diagnosis is associated with improved breast cancer survival, including breast cancer-specific mortality, all-cause mortality, and relapse/metastasis [49]. Linked registry data on prescribed NSAIDs after breast cancer diagnosis and risk of breast cancer recurrence or death show disparate results, with both reduced risks and no associations reported [50–54].

Knowledge regarding the role of breast density and the benefit of NSAIDs for preventing breast cancer is lacking. Given the possible associations between ASA use and reduced breast cancer incidence, prospective randomized trials of ASA treatment among women at high risk of developing breast cancer, such as those with high mammographic density, are warranted. Before such large studies can be performed, pilot studies revealing possible biological effects on the local inflammatory breast microenvironment must be performed.

Here, we show that low-dose ASA (160 mg/day) for 6 months decreased local breast tissue inflammation in healthy postmenopausal women with dense breasts. These changes were breast-specific, as no changes were found in proteins simultaneously sampled from abdominal s.c. fat using the same microdialysis technique. One hypothesis as to why the effects were only detected in breast tissue and not abdominal s.c. fat may be that the breast comprises more diverse cell types and increased number of cells that can contribute to the local inflammatory response compared to s.c abdominal fat. One major advantage of the present study is the use of local in situ sampling of inflammatory proteins rather than monitoring changes in blood or plasma. Most cytokines have a very short half-life and when circulating in blood will rapidly be degraded by other components. Microdialysis enables local sampling and produces very pure specimens, as cells and large enzymes are excluded by the microdialysis membrane. For example, we previously showed that whereas IL-6 and IL-8 levels in plasma are very near the lower detection limits of analytical methods, their levels in microdialysis samples, collected at the same time point, are approximately 1,000 times higher [9]. Therefore, in the present study, we used microdialysis samples of abdominal s.c. fat rather than blood samples to measure systemic effects.

After 6 months of low-dose ASA, we found no differences in breast density, LTF, or PDFF. Although we used a precise and continuous measure of breast density by calculating LTF on MRI as well as PDFF, no changes were observed. This is in line with a previous study using digitized mammographic films, which reported that 325 mg ASA daily for 6 months did not affect breast density [25]. However, the duration of 6 months may be too short, or the magnitude of the decreased inflammatory response may be too small to detect differences in breast density.

By analyzing perfusion characteristics and tissue water relaxation conditions, it is possible to monitor the breast tissue environment and physiology. Contrary to LTF, distinct effects on perfusion in the breast were found after ASA therapy. To some extent, time may affect this parameter, as the rate of contrast in the wash-in phase was decreased in both the no-treatment and ASA groups. However, significantly more women exhibited decreased rates in the ASA group as compared with the no-treatment group. In the wash-out phase, ASA treatment resulted in a prompt decrease in contrast clearance. In addition, levels of the potent angiogenic factor VEGF decreased locally in breast tissue after ASA, and the wash-out rate correlated significantly with VEGF level, supporting the existence of tangible physiological effects of ASA in the breast. Our data are in line with a previous breast cancer study in which a high wash-out fraction was associated with increased microvessel density and angiogenesis, which was in turn associated with an aggressive phenotype [55]. Moreover, aqueous relaxation rates measured using qMRI are related to the microscopic water environment in a range of tissues, for example in multiple sclerosis [56]. Here, we observed a small but significant decrease in water R2 values in fibroglandular tissue across the whole breast only after ASA, suggesting that a more mobile water environment arose as a consequence of treatment.

The strengths of this study were the sophisticated and elaborate investigations of the local breast tissue microenvironment and the randomized approach. Limitations were the relatively small sample size, the unblinded approach and that only postmenopausal women were included.

## Conclusions

We conclude that low-dose ASA for 6 months decreased levels of 20 out of 92 inflammatory proteins in the breast tissue of healthy postmenopausal women with dense breast tissue. Whereas breast density was unaffected by the treatment, breast tissue perfusion rate decreased after ASA. Thus, ASA may shape local breast tissue into an anti-tumorigenic microenvironment. It may be hypothesized that atypical breast epithelial cells have less support from the microenvironment for developing into clinically important breast cancer during ASA therapy. Thus, our results suggest that low-dose ASA may be a relatively nontoxic alternative for breast cancer prevention in postmenopausal women with high mammographic density. Future trials investigating the effects of low-dose ASA and risk of primary breast cancer are warranted.

### Supplementary Information

**Additional file 1. **Characterization of included women and list of proteins included in the analysis.

## Data Availability

Data are available upon reasonable request.

## Abbreviations

ASA: Acetylsalicylic acid
MRI: Magnetic resonance imaging
LTF: Lean tissue fraction
BI-RADS: Breast Imaging Reporting and Data System
ADC: Apparent diffusion coefficient
PDFF: Proton density fat fraction
DWI: Diffusion-weighted images
qMRI: Quantitative relaxometry
MRS: Magnetic resonance spectroscopy
VOI: Volume of interest
AUC: Area under the perfusion curve
FDR: False discovery rate
