# Supplementary material for: Low-dose acetylsalicylic acid reduces local inflammation and tissue perfusion in dense breast tissue in postmenopausal women
Source: Breast Cancer Res. 2024 Feb 5;26:22. doi: 10.1186/s13058-024-01780-2 (PMC10845760; doi:10.1186/s13058-024-01780-2)
Supplement: Supplementary file 1 — Additional file 1. Characterization of included women and list of proteins included in the analysis. [file 13058_2024_1780_MOESM1_ESM.docx]

Supplementary data

**Low-dose acetylsalicylic acid reduces local inflammation and perfusion rate in dense breast tissue in postmenopausal women**

^1,2^Peter Lundberg, ^3^Annelie Abrahamsson, ^2,4^Johan Kihlberg, ^1,2^Jens Tellman, ^4^Ieva Tomkeviciene, ^1^Anette Karlsson, ^2,4^Maria Kristoffersen Wiberg, ^2^Marcel Warntjes, and ^*4^Charlotta Dabrosin

**Table S1.** Characteristics of the included women. Numbers in median (range).

|  | ASA | No treatment | *P*-value |
| --- | --- | --- | --- |
| Age | 65 (55-73) | 66 (56-73) | 0.3 |
| BMI | 23 (19-30) | 24 (19-27) | 0.4 |
| Menopause | 50 (45-60) | 51 (44-57) | 0.2 |
| Menarche | 13 (11-15) | 14 (11-17) | 0.2 |
| Pregnancies | 2 (0-3) | 2 (0-3) | 0.3 |

**Table S2.** The 92 inflammatory that were included in the analyses.

| **Protein** | **Uniprot ID** | **Protein** | **Uniprot ID** | **Protein** | **Uniprot ID** |
| --- | --- | --- | --- | --- | --- |
| 4E-BP1 | Q13541 | FGF-21 | Q9NSA1 | LIF-R | P42702 |
| ADA | P00813 | FGF-23 | Q9GZV9 | MCP-1 | P13500 |
| ARTN | Q5T4W7 | FGF-5 | P12034 | MCP-2 | P80075 |
| AXIN1 | O15169 | Flt3L | P49771 | MCP-3 | P80098 |
| Beta-NGF | P01138 | GDNF | P39905 | MCP-4 | Q99616 |
| CASP-8 | Q14790 | HGF | P14210 | MMP-1 | P03956 |
| CCL11 | P51671 | IFN-gamma | P01579 | MMP-10 | P09238 |
| CCL19 | Q99731 | IL-1 alpha | P01583 | NRTN | Q99748 |
| CCL20 | P78556 | IL-10RA | Q13651 | NT-3 | P20783 |
| CCL23 | P55773 | IL-10RB | Q08334 | OPG | O00300 |
| CCL25 | O15444 | IL-12B | P29460 | OSM | P13725 |
| CCL28 | Q9NRJ3 | IL-15RA | Q13261 | PD-L1 | Q9NZQ7 |
| CCL3 | P10147 | IL-17A | Q16552 | SCF | P21583 |
| CCL4 | P13236 | IL-17C | Q9P0M4 | SIRT2 | Q8IXJ6 |
| CD244 | Q9BZW8 | IL-18R1 | Q13478 | SLAMF1 | Q13291 |
| CD40 | P25942 | IL-20 | Q9NYY1 | ST1A1 | P50225 |
| CD5 | P06127 | IL-20RA | Q9UHF4 | STAMBP | O95630 |
| CD6 | P30203 | IL-22 RA1 | Q8N6P7 | TGF-alpha | P01135 |
| CD8A | P01732 | IL-24 | Q13007 | TNF | P01375 |
| CDCP1 | Q9H5V8 | IL-2RB | P14784 | TNFB | P01374 |
| CSF-1 | P09603 | IL10 | P22301 | TNFRSF9 | Q07011 |
| CST5 | P28325 | IL13 | P35225 | TNFSF14 | O43557 |
| CX3CL1 | P78423 | IL18 | Q14116 | TRAIL | P50591 |
| CXCL1 | P09341 | IL2 | P60568 | TRANCE | O14788 |
| CXCL10 | P02778 | IL33 | O95760 | TSLP | Q969D9 |
| CXCL11 | O14625 | IL4 | P05112 | TWEAK | O43508 |
| CXCL5 | P42830 | IL5 | P05113 | uPA | P00749 |
| CXCL6 | P80162 | IL6 | P05231 | VEGFA | P15692 |
| CXCL9 | Q07325 | IL7 | P13232 |  |  |
| DNER | Q8NFT8 | IL8 | P10145 |  |  |
| EN-RAGE | P80511 | TGF-beta-1 | P01137 |  |  |
| FGF-19 | O95750 | LIF | P15018 |  |  |
|  |  |  |  |  |  |
